# Multi-Omics Characterization of Host-Derived *Bacillus* spp. Probiotics for Improved Growth Performance in Poultry

**DOI:** 10.3389/fmicb.2021.747845

**Published:** 2021-10-20

**Authors:** Dwi Susanti, Alyssa Volland, Nilesh Tawari, Nielson Baxter, Dharanesh Gangaiah, Germán Plata, Akshitha Nagireddy, Troy Hawkins, Shrinivasrao P. Mane, Arvind Kumar

**Affiliations:** ^1^Division of Discovery Biology, Bacteriology and Microbiome, Elanco Animal Health, Greenfield, IN, United States; ^2^Division of Global Computational Sciences, Elanco Animal Health, Greenfield, IN, United States; ^3^Division of Nutritional Health, Elanco Animal Health, Greenfield, IN, United States

**Keywords:** *Bacillus*, probiotics, poultry, growth performance, broiler chickens, multi-omics

## Abstract

Microbial feed ingredients or probiotics have been used widely in the poultry industry to improve production efficiency. Spore-forming *Bacillus* spp. offer advantages over traditional probiotic strains as *Bacillus* spores are resilient to high temperature, acidic pH, and desiccation. This results in increased strain viability during manufacturing and feed-pelleting processes, extended product shelf-life, and increased stability within the animal’s gastrointestinal tract. Despite numerous reports on the use of *Bacillus* spores as feed additives, detailed characterizations of *Bacillus* probiotic strains are typically not published. Insufficient characterizations can lead to misidentification of probiotic strains in product labels, and the potential application of strains carrying virulence factors, toxins, antibiotic resistance, or toxic metabolites. Hence, it is critical to characterize in detail the genomic and phenotypic properties of these strains to screen out undesirable properties and to tie individual traits to clinical outcomes and possible mechanisms. Here, we report a screening workflow and comprehensive multi-omics characterization of *Bacillus* spp. for use in broiler chickens. Host-derived *Bacillus* strains were isolated and screened for desirable probiotic properties. The phenotypic, genomic and metabolomic analyses of three probiotic candidates, two *Bacillus amyloliquefaciens* (*Ba* ATCC PTA126784 and ATCC PTA126785), and a *Bacillus subtilis* (*Bs* ATCC PTA126786), showed that all three strains had promising probiotic traits and safety profiles. Inclusion of *Ba* ATCC PTA12684 (*Ba*-PTA84) in the feed of broiler chickens resulted in improved growth performance, as shown by a significantly improved feed conversion ratio (3.3%), increased of European Broiler Index (6.2%), and increased average daily gain (ADG) (3.5%). Comparison of the cecal microbiomes from *Ba PTA*84-treated and control animals suggested minimal differences in microbiome structure, indicating that the observed growth promotion presumably was not mediated by modulation of cecal microbiome.

## Introduction

An increasing demand for poultry meat and egg has put significant pressure on the poultry industry to improve production efficiency. The Food and Agriculture Organization of the United Nations (FAO) projected that global meat and egg consumption will increase by 52 and 39%, respectively, in 2050 compared to 2012 (FAO-UN, 2018). The challenge is further heightened by restrictions from European Union and United States regulatory bodies on the use of antibiotics as growth promoters (AGPs) and prophylactic care; this change is due to public health concerns related to the development and spread of antibiotic resistant bacteria (US-FDA, 2013; Koutsoumanis et al., 2020). Antibiotics have been used as AGPs for over half a century, aiding growth performance and controlling disease outbreaks (Moore et al., 1946).

For the above reasons, microbial feed ingredients, also called direct-fed microorganisms (DFMs) or probiotics, have attracted tremendous interest as an alternative to AGPs to support improved production efficiency. Probiotics are defined as “live microorganisms that, when administered in adequate amounts, confer a health benefit on the host” (Hill et al., 2014). Probiotics are believed to exert their benefits through the following proposed mechanisms: assisting with nutrition and digestion, competitive exclusion of pathogens, modulating the immune system and gut microbiota, improving epithelial integrity, and/or producing small molecule metabolites that are beneficial to the host (Grant et al., 2018; Jha et al., 2020). In addition to the above probiotic effects, microorganisms used as probiotics must survive environmental and processing challenges prior to reaching their target site. This includes low acidity of the upper gastrointestinal tract (GIT), bile acid toxicity, and heat exposure during feed pelleting application, which could pose challenges to the use of traditional probiotic strains such as those belonging to the genera *Lactobacillus* and *Bifidobacterium*, as they are often sensitive to some of these extreme conditions.

Endospore-forming *Bacillus* spp. offer advantages over traditional probiotic strains due to the ability of *Bacillus* spores to withstand hostile environments such as high temperature, desiccation, and acidic pH, resulting in increased viability during the manufacturing and feed-pelleting process, increased stability inside animals’ GIT and extended product shelf-life. Thus, *Bacillus* strains have been widely used to support improved production parameters (Barbosa et al., 2005; Guo et al., 2006; Setlow, 2006; Shivaramaiah et al., 2011). *Bacillus* spp., commonly found in soil, enter the animal GIT through feed or ingestion of fecal material. Once inside the GIT, the spores germinate into metabolically active vegetative cells, eliciting their probiotic effect (Hoa et al., 2001; Casula and Cutting, 2002; Latorre et al., 2014; Bernardeau et al., 2017). Within the *Bacillus* genus, species commonly used as probiotics are *Bacillus subtilis, Bacillus coagulans, Bacillus clausii, Bacillus amyloliquefaciens*, and *Bacillus licheniformis* (Mingmongkolchai and Panbangred, 2018). *Bacillus* strains are known to produce commercial enzymes, antimicrobial peptides, and small metabolites that may confer health benefits to the host by supporting improved feed digestion, suppressing undesirable organisms, and by maintaining a healthy gut microbiota and immune system [reviewed in Grant et al. (2018)].

Despite several reports on the benefits of *Bacillus* spores as probiotics and DFMs on human and animal health, respectively, detailed characterizations of *Bacillus* spp. strains to not only help explain their potential probiotic properties but also to evaluate their safety are typically unavailable in the public domain. Insufficient characterization of probiotic strains could result in the following undesirable outcomes: (1) misleading identification at the genus and species levels of probiotic strains in commercial products; and (2) the occurrence of antimicrobial resistance traits and toxins in probiotic strains that could negatively impact the host and raise public health concerns. As an example of the first instance, commercial probiotics labeled as *B. coagulans* were later shown to be *Lactobacillus sporogenes*, and *B. clausii*-containing probiotics were mislabeled as comprised of *B. subtilis* (Green et al., 1999; Weese, 2002; Weese and Martin, 2011).

To fill the knowledge gap in the genomic and phenotypic characterization of *Bacillus* spp. DFMs, we take advantage of DNA sequencing and omics technologies for the comprehensive identification, screening, and characterization of *Bacillus* spp. to assess their safety and efficacy as probiotic candidates. Detailed strain characterizations employing multi-omics approaches could uncover correlations between strain properties and the effects of probiotic administration on the host, underpin possible mechanisms of action of probiotic strains, identify biomolecules that could be used in place of live bacteria (i.e., peptides, enzymes, and metabolites), and help to rationally design strain consortia to maximize positive effects on the host.

Here, we present a comprehensive screening and multi-omics characterization for *Bacillus* spp. as probiotics for use in poultry. Our analysis provides insights into the genotypic, phenotypic, and metabolomic properties of three *Bacillus* spp. strains that show desirable probiotic traits and safety profiles. Furthermore, results from a clinical study of the administration of one of the strains -*B. amyloliquefaciens* Ba PTA84- showed a significant improvement of broiler growth performance. Such in-depth characterization and the data made available will guide future efforts to develop next generation probiotics, microbial-derived nutritional health products, and inform decisions to design microbial consortia for the potential improvement of poultry production efficiency.

## Materials and Methods

### Microbial Strains and Growth Conditions

The *Bacillus* spp. strains were routinely grown in Lysogeny Broth (LB) and incubated at 37°C overnight while shaking at 200 rpm. Avian pathogenic *Escherichia coli* (APEC) serotypes O2, O18, O78, and *Clostridium perfringens* NAH 1314-JP1011 were obtained from the Elanco pathogen library. *Salmonella enterica* serovar Typhimurium ATCC 14028 was purchased from the American Type Culture Collection (ATCC, Manassas, VA, United States). *E. coli* strains and *S*. Typhimurium, were routinely grown in LB, and *C. perfringens* was grown in anaerobic Brain Heart Infusion (BHI) broth supplemented with yeast extract (5.0 g/L) and L-cysteine (0.5 g/L). For growth in liquid culture, a colony from the respective agar plate was inoculated into a 10 mL tube containing liquid media and the tube was incubated in a shaker incubator at 37°C and 200 rpm for *E. coli* and *S*. Typhimurium, and statically at 39°C for *C. perfringens* inside a Bactron anaerobic chamber (Sheldon Manufacturing, Inc., Cornelius, OR, United States). The anaerobic chamber contained a mixture of N_2_:CO_2_:H_2_ (87.5:10:2.5, v/v/v).

### Vero Cells Growth Condition

Vero cells were obtained from Elanco cell culture collection and were maintained in Opti-MEM^®^ I reduced serum media containing 5% Fetal Bovine Serum (FBS) (Cytiva, Marlborough, MA, United States) and Gentamicin (Opti-5-Gent) (Life Technologies, Carlsbad, CA, United States). The serum-free cell culture medium was similarly prepared with Minimal Essential Medium with Earle’s Balanced Salt Solution (MEM/EBSS), 10% fetal bovine serum (FBS), 1% non-essential amino acids and 1% L-glutamine in place of FBS. To generate wells containing 100% confluent cells for the cytotoxicity assay, Vero cells grown for 2–3 days were divided into a 96-well flat bottom tissue culture plate (Fisher Scientific, Waltham, MA, United States) where each well contained 1 × 10^4^ cells. The cells were then incubated on the plate for 48–72 h inside a CO_2_ incubator (37^*o*^C; %CO_2_ was maintained at 5 ± 1%).

### *Bacillus* spp. Isolation and Identification

#### *Bacillus* Isolation

*Bacillus* spp. were isolated from cecal contents of healthy 30–42 day old chickens raised at poultry research farms in Arkansas, Georgia, and Indiana, United States employing a combination of a high-throughput isolation platform employing Prospector^®^ (GALT, Inc., San Carlos, CA, United States) following the manufacture’s protocol, and a classical isolation method as described previously (Koransky et al., 1978). For both approaches, isolation protocols were preceded by selection of *Bacillus* spores from the starting cecal contents by applying heat at 95°C for 5 min or treatment with ethanol. For the latter, frozen cecal samples from the Elanco library preserved in BHI containing 20% glycerol were thawed and equal amounts of Tryptic Soy Broth (TSB) medium were added and mixed. An equal amount of absolute ethanol was added to the sample to a final concentration of 50% and the mixture was incubated at 30°C for an hour. The ethanol-treated samples were then used for isolation. For *Bacillus* spp. isolation employing conventional methods, 10-fold serial-dilution was applied to the treated cecal samples to ensure separate colonies recovered on agar plates. Each colony was purified by three sequential passages onto agar plates.

#### Strain Identification

For an initial strain identification, *Bacillus* cell lysates were sent to the TACGen genomic sequencing facility (Richmond, CA, United States) for strain identification. The strain identities were determined by Sanger sequencing of amplified regions of a partial length of 16S ribosomal RNA (rRNA) gene employing primers 27F (5′ AGA GTT TGA TCM TGG CTC AG 3′) and 1492R (5′ CGG TTA CCT TGT TAC GAC TT3′). The resulting 16S rRNA sequences were then searched against the NCBI 16S rRNA database using BLAST searches with an *e*-value cutoff of <10^–20^ and a percent sequence identity value of >95%. Strain identification of select isolates were further confirmed by ortholog analyses as described in the following section “Genome-Based Strain Identification and Comparative Genomic Analyses”.

### *In vitro* Microorganism Inhibition Assay

Eight *Bacillus* spp. strains were screened for their antimicrobial activity against five microorganisms, namely APEC serotypes O2, O18, O78, *Salmonella* Typhimurium ATCC 14028, and *C. perfringens* NAH 1314-JP1011. The assays were modified from a protocol described in Latorre et al. (2016) and performed in duplicate. The detailed protocol is presented in Supplementary Materials.

### Enzyme Activities

The β-mannanase assay was adapted from a protocol as described by McCleary (1978). Assays for amylase and protease activities were done following protocols in Latorre et al. (2016). These protocols are presented in Supplementary Materials.

### Cytotoxicity Assay

Cytotoxicity assays of eight *Bacillus* culture supernatants were performed following the protocol described in EFSA guidelines (EFSA Panel on Additives and Products or Substances used in Animal Feed [FEEDAP] et al., 2018). A detailed protocol is described in Supplementary Materials. Culture supernatant of *B. cereus* ATCC 14579 and *B. licheniformis* ATCC14580 were used as positive and negative controls, respectively.

### Antimicrobial Susceptibility Assessment

Antibiotic susceptibility assays of *Bacillus* spp. for tetracycline, chloramphenicol, streptomycin, kanamycin, erythromycin, vancomycin, gentamycin, ampicillin, and clindamycin were performed and assessed according to an EFSA guideline for Antimicrobial resistance of the *Bacillus* spp. as direct fed microbials (DFM) (EFSA Panel on Additives and Products or Substances used in Animal Feed [FEEDAP] et al., 2018). *Bacillus* spp. strains on LB agar plates were sent to Microbial Research, Inc. (Fort Collins, CO, United States) for analysis following protocols in compliance with Clinical Laboratory Standard Institute (CLSI) document VET01 (World, 2021). Briefly, minimum inhibitory concentration (MIC) plates were prepared using cation-adjusted Mueller Hinton Broth (MHB) and the antimicrobials were twofold serially diluted to obtain a final concentration range of 0.06–32 μg/mL. Growth of *Bacillus* spp. in the presence of each of nine antimicrobials with different dilutions was monitored. Susceptibility was interpreted as the lack of *Bacillus* spp. growth in the presence of antimicrobial at a concentration that was lower that the cut-off values of the respective antimicrobials described in the EFSA guideline. For quality control, the following organisms were used as controls, *E. coli* ATCC 25922, *Enterococcus faecalis* ATCC 29212, *Pseudomonas aeruginosa* ATCC 27853 and *Staphylococcus aureus* ATCC 29213.

### Whole Genome Sequencing, Assembly, and Annotation

#### Genomic DNA Isolation

High molecular weight genomic DNA of *Bacillus* spp. were extracted employing a Phenol:Chloroform:Isoamyl alcohol (PCI) method as described previously (Rao et al., 2018). Bacterial cells were harvested by centrifugation at 7,000 × *g* for 10 min from an overnight culture of *Bacillus* spp. grown in 25 mL LB supplemented with 0.005% Tween 80 in 50 mL sterile Falcon tube (Fisher Scientific, Waltham, MA, United States). The resulting cell pellet was resuspended in 0.75 mL of 1X Tris-EDTA (TE) buffer (Life Technologies, Carlsbad, CA, United States), pH 8, containing Tris-HCl and EDTA at final concentrations of 10 and 1 mM, respectively, in a 2 mL Eppendorf tube (Fisher Scientific, Waltham, MA, United States). To lyse the cells, Lysozyme (Sigma Aldrich, St. Louis, MO, United States) was added at a final concentration of 7 mg/mL and the mixture was incubated at 37°C for an hour. Then, SDS and Proteinase K (Sigma-Aldrich, St. Louis, MO, United States) were added to the mixture at final concentrations of 2% and 400 μg/mL, respectively, and the lysate was incubated at 60°C for 1 h. To remove RNA from the cell lysate, 10 μL of RNase (Thermo Fisher Scientific, Waltham, MA, United States) were added and the mixture was incubated at 37°C for 30 min. An equal volume of a mixture of PCI (25:24:1, v/v/v) was added to the supernatant and was mixed by carefully inverting tubes 5–10 times rigorously. The aqueous phase containing DNA was separated from the organic phase by centrifugation at 12,000 × *g* for 15 min, and the top aqueous layer was collected into a fresh 2 mL Eppendorf tube. An equal volume of a mixture of Chloroform:Isoamyl alcohol (24:1, v/v) was added to this aqueous phase containing DNA, and mixed by carefully inverting the tube. The mixture was centrifuged at 12,000 × *g* for 10 min. DNA from the aqueous layer was precipitated by an addition of one tenth volume of sodium acetate (3 M, pH 5.2) followed by centrifugation at 16,000 × *g* for 20 min. The DNA pellet was washed three times with ice-cold 70% ethanol, air-dried, and resuspended in 0.5 mL 1X TE buffer.

#### PacBio Long Read Genome Sequencing

The bacterial genomic DNA samples were shipped on dry-ice to DNA Link, Inc. (San Diego, CA, United States) for whole genome sequencing using PacBio RSII platform. Briefly, 20 kb DNA fragments were generated by shearing genomic DNA using the covaris G-tube according to the manufacturer’s recommended protocol (Covaris, Woburn, MA, United States). Smaller fragments were purified by the AMpureXP bead purification system (Beckman Coulter, Brea, CA, United States). For library preparation, 5 μg of genomic DNA were used. The SMRTbell library was constructed using SMRTbell^TM^ Template Prep Kit 1.0 (PacBio^®^, Menlo Park, CA, United States). Small fragments were removed using the BluePippin Size selection system (Sage Science, Beverly, MA, United States). The remaining DNA sample was used for large-insert library preparation. A sequencing primer was annealed to the SMRTbell template and DNA polymerase was bound to the complex using DNA/Polymerase Binding kit P6 (PacBio^®^, Menlo Park, CA, United States). Following the polymerase binding reaction, the MagBead was bound to the library complex with MagBeads Kit (PacBio^®^, Menlo Park, CA, United States). This polymerase-SMRTbell-adaptor complex was loaded into zero-mode waveguides. The SMRTbell library was sequenced by 2 PacBio SMRT cells (PacBio^®^, Menlo Park, CA, United States) using the DNA sequencing kit 4.0 with C4 chemistry (PacBio^®^, Menlo Park, CA, United States). A 1 × 240-min movie was captured for each SMRT cell using the PacBio RS sequencing platform.

#### Genome Assembly, Annotation and Features Prediction

The genome was assembled by DNA link, Inc. with HGAP.3. Genome annotation was carried out using a custom annotation pipeline by combining several prediction tools. Coding sequences, transfer RNA and transmembrane RNA were predicted and annotated using Prokka (Laslett and Canback, 2004; Hyatt et al., 2010; Seemann, 2014). Ribosomal binding site (RBS) prediction was carried out using RBSFinder (Suzek et al., 2001). TranstermHP was used to predict Rho-independent transcription terminators (TTS) (Kingsford et al., 2007). Ribosomal RNA and other functional RNAs such as riboswitches and non-coding RNA was annotated with Infernal (Nawrocki et al., 2009). Operons were predicted based on primary genome sequence information with Rockhopper v2.0.3 using default parameters (Tjaden, 2020). Insertion sequence prediction was done using ISEscan v.1.7.2.1 (Xie and Tang, 2017). Prophage prediction was done using PhiSpy v4.2.6 which combines similarity- and composition-based strategies (Akhter et al., 2012).

### Genome-Based Strain Identification and Comparative Genomic Analyses

Taxonomic labeling of the assembled microbial genomes was carried out using CAMITAX (Bremges et al., 2020). CAMITAX is a scalable workflow that combines genome distance–, 16S ribosomal RNA gene–, and gene homology–based taxonomic assignments with phylogenetic placement. OrthoFinder v2.3.1 (Emms and Kelly, 2019) was used to determine orthologous relationships (Sayers et al., 2021).

### Phylogenetic Analysis

Phylogenetic relationships of the genomes were explored with UBCG v3.0 using default settings (Na et al., 2018). This software tool employs a set of 92 single-copy core genes commonly present in all bacterial genomes. These genes then were aligned and concatenated within UBCG using default parameters. The estimation of robustness of the nodes is done through the gene support index (GSI), defined as the number of individual gene trees, out of the total genes used, that present the same node. A maximum-likelihood phylogenetic tree was inferred using FastTree v.2.1.10 with the GTR + CAT model (Price et al., 2010).

### Patent Depository of *Bacillus amyloliquefaciens* ATCC PTA-126784 and PTA-126785, and *B. subtilis* ATCC PTA-126786

*Bacillus amyloliquefaciens* ATCC PTA-126784 and PTA-126785, and *B. subtilis* ATCC PTA-126786 strains were deposited in the ATCC culture collection (Manassas, VA, United States). For simplicity, *Bacillus amyloliquefaciens* ATCC PTA-126784 and PTA-126785, and *B. subtilis* ATCC PTA-126786 strains are referred to as *Ba* PTA84 and *Ba* PTA85, and *Bs* PTA86, respectively, throughout the manuscript.

### Global Untargeted Metabolomic Analysis

*Bacillus* strains *Bs* PTA86, *Ba* PTA84, and *Ba* PTA85 were grown as three single strain cultures, and then were analyzed as a two-strain (*Ba*-PTA84 and PTA85) or three-strain (*Bs*-PTA86, *Ba*-PTA84, and *Ba*-PTA85) consortia in 5 mL of minimal or rich liquid media. For growth in minimal media, medium containing 1X M9 salts, and glucose at a final concentration of 0.5% (w/v) was used. Rich medium contained the following entities (g/L): peptone 30; sucrose 30; yeast extract 8; KH_2_PO_4_ 4; MgSO_4_ 1; and MnSO_4_ 0.025. The culture was grown at 37°C overnight. *Bacillus* cells were pelleted by centrifugation at 10,000 × *g* for 10 min, cell pellets were washed three times with ice-cold PBS. The resulting cell pellets and cell-free supernatants were stored at −80°C and sent to metabolon Inc. (Durham, NC, United States) for global untargeted metabolomic profiling. The experiment was performed in one biological replicate. Detailed description of metabolomic analysis is presented in Supplementary Materials.

### *In vivo* Assessment of *Bacillus* DFM for Improvement of Growth Performance in Broiler Chickens

#### Spore Generation

*Bacillus* spores were generated employing a modified protocol as described in Setlow (1990). *Bacillus* spp. was grown in a liquid Difco sporulation medium containing Nutrient Broth (BD Difco, Franklin Lakes, NJ, United States), 8.0 g/L; KCl, 1g/L, and MgSO_4_⋅7H_2_O, 0.12 g/L. The mixture was adjusted to pH 7.6 with additions of NaOH. After adjusting the pH and sterilizing the media by the use of an autoclave at 121°C, 1 mL of each of the following mineral sterile stock solutions were added to broth media, 1.0 M CaCl_2,_ 0.01 M MnSO_4_, 1.0 mM FeSO_4_. A sterile glucose solution was also added to the medium mixture to a final concentration of 5.0 g/L. A single colony was taken from an agar plate and was inoculated into 100 mL of the sporulation medium. The culture was incubated overnight at 37°C with shaking at 200 rpm. This culture served as a seeding culture for 1 L of liquid culture. All growth were done employing vented baffled flasks. This culture was incubated at 37°C while shaking at 200 rpm for at least 72 h. The presence of spores was monitored with a brightfield microscope. The spores were harvested at 17,000 rpm and washed three times with pre-chilled sterile distilled water. The spores were then resuspended in 30 mL of pre-chilled sterile distilled water and the spore suspension was mixed with irradiated ground rice hulls (Rice Hull Specialty Products, Stuttgart, AR, United States), dried at 60°C for 3–4 h to eliminate vegetative cells. To determine spore inclusion in the rice hulls, 0.25 g of the material containing spores was heat treated at 90°C for 5 min. One milliliter of water was added to the material and allowed to soak for 15–30 min. The suspension was vortexed for 30 s and serially diluted 10-fold for colony counts on agar plates.

#### Study Design

A total of 2,500 1-day-old male broiler chicks (Cobb 500) were randomly allocated to two treatment groups on Study Day (SD) 0. The control group received only the basal diet, while the treated group received the basal diet plus 1.5 × 10^5^ CFU of *Ba* PTA84 per gram of final feed. The control group consisted of 30 pens of 50 birds per pen, and the *Ba* PTA84 group consisted of 20 pens of 50 birds per pen.

Birds were housed in floor pens in a single environmentally controlled room with *ad libitum* access to treatment diets and water. Basal diets were formulated to be iso-nutritive, and to meet or exceed the nutrient requirements recommended for broilers (Supplementary Table 1). Feed was issued in four study phases: Starter Phase I (SD 0–12); Grower Phase II (SD 12–26); Finisher Phase III (SD 26–35), Withdrawal Phase IV (SD 35–42). Diets did not contain antibiotics, anticoccidials or growth promoters and were fed to the birds as a mash in all phases.

Bird weights (pen weight) were measured and recorded at SD 0, 12, 26, 35, and 42. Feed issued and weighed back were recorded for each feeding phase. Bird general health, mortality and environmental temperature were recorded daily.

#### Statistical Analysis

The experimental unit was the pen. All statistical analysis was conducted using the SAS system version 9.4 (SAS Institute, Cary, NC, United States) and all tests were performed comparing the control group to the treated group using a one-sided test at *P* < 0.05 level of significance.

Performance variables of interest for each feeding period and overall included: live final body weight (LFBW), average daily gain (ADG), average daily feed intake (ADFI), gain to feed efficiency (GF), feed to gain efficiency (FCR), mortality, and the European Broiler Index (EBI). These variables were calculated and evaluated for each study phase [Starter, Grower, Finisher, Withdrawal and Overall (SD 0–42)] and both adjusted for mortality and unadjusted.

### Microbiome Profiling of Cecal Content From Birds Treated With *Ba* PTA84

#### DNA Extraction, Library Preparation and Sequencing

Total DNA from cecal content samples, collected from 20 animals from each *Bacillus*-fed and control group, were extracted employing the Lysis and Purity kit (Shoreline Biome, Farmington, CT, United States) following manufacturer’s protocol. The resulting DNA was used as template for library preparation using Shoreline Biome’s V4 16S DNA Purification and Library Prep Kit (Shoreline Biome, Farmington, CT, United States). Briefly, PCR amplification of the V4 region of the 16S rRNA gene was performed using the extracted DNA and the primers 515F (5′GTGGCCAGCMGCCGCGGTAA) and 806R (5′-GGACTACHVHHHTWTCTAAT). The resulting amplicons were then sequenced using 2 bp × 150 bp paired-end kits on the Illumina iSeq platform. To increase diversity, PhiX 50 pM was added to a final concentration of 5% into the amplicon library.

#### Bioinformatic Analysis

Forward and reverse reads were processed with cutadapt (v 2.5) (Martin, 2011) to remove primer sequences. Read pairs without primer sequences present or more than 15% primer mismatches were discarded. The DADA2 pipeline (v. 1.12.1) (Callahan et al., 2016) was used to generate a count matrix of amplicon sequence variants (ASVs) across samples. Due to the short length of iSeq reads, forward and reverse reads were trimmed to a length of 110 bp and merged with DADA2’s *justConcatenate* option. The DADA2 parameters parameters *maxN* = *0, truncQ* = *2, rm.phix* = *TRUE* and *maxEE* = *2* were used. Taxonomic labels were assigned to each ASV using the DADA2 *assignTaxonomy* method and the Silva v. 138 database (Yilmaz et al., 2014). Diversity and richness per sample were quantified from the ASV matrix using the Simpson, Shannon and Chao indices (Simpson, 1949; Chao, 1984; Shannon, 1997) and compared across treatments with the Mann–Whitney *U* test. Comparison of microbiome structures across treatments was performed using PERMANOVA and ANOSIM analysis based on the Bray–Curtis dissimilarity between samples. PERMANOVA and ANOSIM were performed using code in the scikit-bio python package^1^. Principal component analysis of the Bray–Curtis dissimilarity matrix was used to analyze sample clustering according to treatment group.

## Results

### Isolation and Identification *Bacillus* spp. From Healthy Animals

*Bacillus* spp. strains were isolated from the cecal contents and fecal materials of healthy chickens. The taxonomic identities of the isolates were determined by 16S-rRNA amplicon sequencing. These isolates belonged to 30 different *Bacillus* species with the top hits of *B. velezensis, B. amyloliquefaciens*, *Bacillus haynesii, Bacillus pumilus, B. subtilis*, and *B. licheniformis*, Supplementary Table 2.

Due to safety considerations, *Bacillus* spp. isolates chosen for further screening included only those that belong to the species listed as DFMs in the Association of American Feed Control Officials, Inc. (AAFCO) Official Publication since they “were reviewed by FDA Center for Veterinary Medicine and found to present no safety concerns when used in direct-fed microbial products” (AAFCO, 2018), and to the species listed as Qualified Presumption of Safety (QPS) status according to the European Food Safety Authority (EFSA) BIOHAZ Panel (Koutsoumanis et al., 2020). These were *B. subtilis*, *B. amyloliquefaciens*, *B. pumilus*, and *B. licheniformis*.

### *In vitro* Screening for Probiotic Properties of *Bacillus* spp. Strains

**FIGURE 1:**
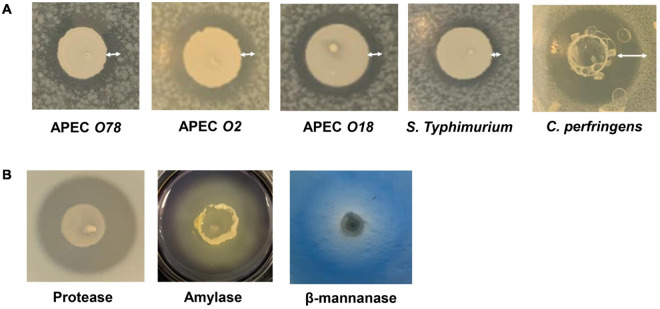
Screening of *Bacillus* spp. DFM candidates for pathogen growth inhibition and enzyme activities. **(A)** Microorganism growth inhibition. Representative examples of microorganism overlay assays were presented with the following microorganisms, Avian Pathogenic *Escherichia coli* serotype O78, O2, and O18, *Salmonella enterica* serovar Typhimurium ATCC 14028, and *Clostridium perfringens* NAH 1314-JP1011. Overlay microorganism inhibition assays were performed in duplicate for each of the *Bacillus* spp. strains. A zone of inhibition was measured from the edge of *Bacillus* spp. until the end of clearance zone as shown as white double-edge arrows. **(B)** Screening of digestive enzyme activities of *Bacillus* spp. strains. The assay was performed in duplicate for each *Bacillus* spp.

*Bacillus* spp. strains were tested to determine their effect on selected microorganisms and their ability to secrete selected enzymes (Latorre et al., 2016). For the former, Gram-negative and Gram-positive microorganisms (*E. coli* O2, O18, O78, *C. perfringens* NAH 1314-JP1011, and *S. enterica* serovar Typhimurium ATCC 14028) were used. For the latter, plate-based assays for determining the secretion of amylase, protease, and β-mannanase were performed (Figure 1).

A total of 266 *Bacillus* strains were first screened against *E. coli* O2, and 71% of the strains showing positive *E. coli* O2 inhibition were selected for a second-round of assays targeting *E. coli* O18, then *E. coli* O78, *S.* Typhimurium and lastly *C. perfringens* JP1011. The top 8 *Bacillus* strain candidates were selected according to their cumulative inhibition scores, Supplementary Table 3. These included 6 *B. amyloliquefaciens* (*Ba*): *Ba* ELA006, *Ba* ELA071, *Ba* ELA151, *Ba* ELA175, *Ba* PTA84, *Ba* PTA85, and 2 *B. subtilis* (*Bs*) isolates; *Bs* ELA082 and *Bs* PTA86.

The cumulative inhibition score was calculated as the sum of the inhibition score values of a *Bacillus* strain against the five microorganisms tested. The data showed that six *Ba* strains had better cumulative microbial inhibition scores compared to two *Bs* strains. The average cumulative inhibition scores of 8.5 and 5.5 for six of *Ba* and two of *Bs*, respectively.

The eight *Bacillus* strain candidates were also evaluated for their ability to secrete enzymes. *Bacillus* strains are known to produce a variety of enzymes (Contesini et al., 2018; Danilova and Sharipova, 2020). *In vitro* plate-based assays for protease, amylase, and β-mannanase activities showed that six *Ba* and *two Bs* showed comparable amylase, protease, and β-mannanase activities, Supplementary Table 3 *Ba* ELA071 and *Ba* PTA85 showed the lowest and highest cumulative REA values of 4.73 and of 6.1, respectively.

### Safety Assessment of *Bacillus* spp. Probiotic Candidates

To evaluate the safety of *Bacillus* spp. as microbial feed ingredients, the *Bacillus* candidates were tested for antimicrobial susceptibility to medically relevant antimicrobials.

**FIGURE 2:**
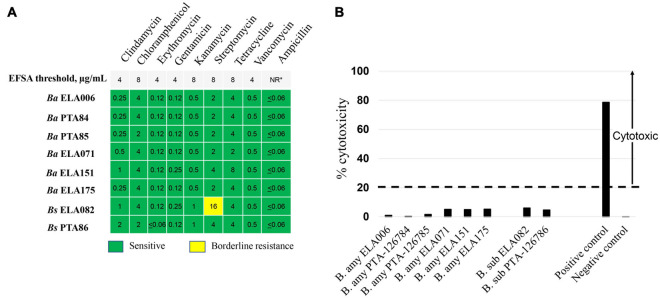
A safety assessment of *Bacillus* spp. DFM candidates. **(A)** Antimicrobial susceptibility tests of *Bacillus* spp. MIC (μg/mL) values for each antibiotic tested of respective *Bacillus* spp. were shown. Nine medically important antibiotics at a concentration range of 0.06–32 μg/mL were tested and the respective antimicrobial susceptibility cut-off concentrations required for *Bacillus* spp. were shown at the top panel. **(B)** Cytotoxicity assessment of *Bacillus* spp. culture supernatant toward Vero cells. Culture supernatant of *B. cereus* ATCC 14579 and *B. licheniformis* ATCC14580 were used as positive and negative controls, respectively. Cytotoxicity level above 20%, dashed line, is considered cytotoxic. *, NR, not required by EFSA guidelines.

The results from antimicrobial susceptibility tests for 8 *Bacillus* strains showed that all strains were susceptible to the tested antibiotics. The only exception was *Bs* ELA082 which exhibited a borderline resistance to streptomycin. The MIC of streptomycin for this strain was 16 μg/mL, which is twofold higher than the EFSA threshold cut-off of streptomycin for *Bacillus* as DFM, Figure 2A.

To determine the potential toxicity of *Bacillus* strains on the host cells, culture supernatants of *Bacillus* spp. were tested for cytotoxicity toward Vero cells according to EFSA Panel on Additives and Products or Substances used in Animal Feed [FEEDAP] et al. (2018). The cytotoxicity assay was performed by monitoring the lactate dehydrogenase (LDH) enzyme originated from compromised Vero cells as described in Fagerlund et al. (2008). Figure 2B shows the cytotoxicity levels of each *Bacillus* strain tested. The results suggested that all 8 *Bacillus* strains were non-cytotoxic with toxicity levels far below 20%, a percentage that is considered cytotoxic according to the EFSA guidelines. Of all isolates, the cytotoxicity level of *Ba* PTA84 was closest to that of the negative control. In general, *Ba* strains exhibited toxicity levels that were lower than those of *Bs* strains. Within the *B. subtilis* group, the cytotoxicity level of *Bs* PTA86 was the lowest, 5%.

### Selection of *Bacillus* spp. as Direct Fed Microbial Candidates

Based on their performance on microorganism inhibition, enzymatic activities, antimicrobial susceptibility, and low toxicity against Vero cells, the strains *Ba* PTA84, *Ba* PTA85, and *Bs* PTA86 were chosen for more detailed characterization employing genomic and metabolomic approaches described in the following sections.

### Untargeted Global Metabolomic Analysis of Cell Pellets and Culture Supernatants of *Ba* PTA84, *Ba* PTA85, and *Bs* PTA86 as Single Strains and as Consortia

Untargeted metabolomics analysis of cell pellets and culture supernatants of the three candidate strains was performed to assess metabolite profiles of each strain and strains-in consortia when grown in two different media. Cells were cultured in both rich and minimal media as individual strains, as well as a consortium of *Ba* PTA84 and *Ba* PTA85, and a consortium including all three strains. In total, 436 and 457 named metabolites were identified in the supernatant and pellet samples, respectively (Supplementary Tables 4–7).

**FIGURE 3:**
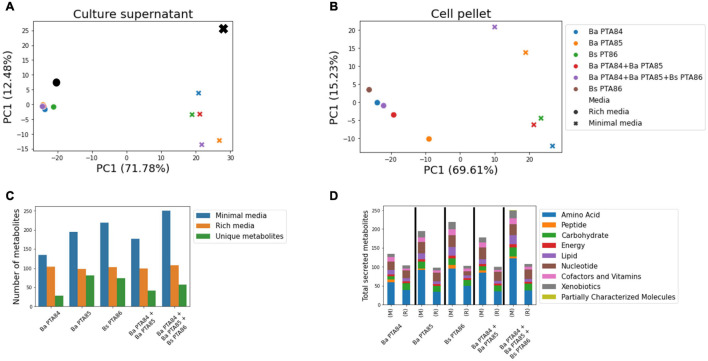
Global untargeted metabolomic analysis of *Ba* PTA84, *Ba* PTA85, and *Bs* PTA86. **(A)** Principal component analysis of scaled metabolite intensities for culture supernatants in two different media. Black symbols represent media controls; numbers in parentheses indicate the variance explained by the first two principal components. **(B)** Like **(A)**, but for the culture cell pellets. **(C)** Number of identified secreted metabolites in culture supernatants in two different media (M) and (R), minimal and rich media, respectively. Secreted metabolites were defined as having a scaled intensity at least 1.5-fold higher than observed in media controls. Unique metabolites represent secreted compounds with abundances at least 1.5-fold higher than observed for other single strains (in the case of individual strain cultures) or corresponding individual strains (in the case of co-cultures). **(D)** Pathway representation of metabolites secreted by strains or strain combinations under minimal and rich media culture conditions.

A principal component analysis (PCA) of the normalized metabolite abundances showed a clear separation between samples from rich and minimal media along the first principal component (∼70% explained variance), both in the supernatant and pellet (Figures 3A,B). Additionally, the separation between samples along the two first principal components suggested that under the tested growth conditions the strains and consortia differ in their profiles of secreted/consumed metabolites as well as their cell-pellet small-molecule content.

Interestingly, looking at the metabolite profiles in the culture supernatant (Figure 3A), all strains and strain combinations tightly clustered in the rich media samples while they separated under minimal media conditions. Indeed, looking at the number of secreted metabolites under each condition (abundance > 1.5-fold above media controls, Figure 3C), cells in all cultures secreted approximately 100 named metabolites in rich media with a median Jaccard similarity of 0.8, compared to a range of 134–250 secreted metabolites in minimal media with only 0.54 median overlap (Mann–Whitney *p*-value = 0.01). This shows that especially under minimal media conditions, each strain or consortia secretes distinct sets of small molecules.

In rich media, *Ba* PTA84 secreted the largest number of metabolites (104 metabolites) and it had the fewest uniquely secreted metabolites (abundance > 1.5-fold higher than other individual strains) across both media. Thirteen unique metabolites related to amino acid, central carbon, nucleotide, and lipid metabolisms as well as some xenobiotic compounds (i.e., quinate, 4-hydroxybenzyl alcohol, and 3-dehydroshikimate) were detected.

As expected, the three-strain consortium was found to have the largest number of secreted metabolites in minimal media, a total of 250 metabolites. These include potential host beneficial metabolites such as betaine, kynurenine, indolactate, tyrosol, citrulline, tricarballylate, vitamins B5 and B6, hippurate, and kestose. Of all three single strains, the culture supernatant of *Bs* PTA86 carried the highest number of metabolites in minimal media (219 metabolites), followed by *Ba* PTA85 and *Ba* PTA84 with 195 and 130 metabolites, respectively. *Ba* PTA85 had the greatest number of unique secreted metabolites, a total 78 metabolites. The larger number of secreted metabolites under minimal media conditions was partly due to a higher number of amino acid metabolism intermediates, followed by nucleotide and carbohydrate metabolites (Figure 3D). Thus, our results suggest that the different strains may use different metabolic strategies to synthesize amino acids/proteins, nucleotide, and carbohydrate molecules given a limited nutrient availability.

### Genome Properties of *Ba* PTA84 and PTA85, and *Bs* PTA86

The genomes of *Ba* PTA84, *Ba* PTA85, and *Bs* PTA86 were sequenced by PacBio sequencing. Assembly of *Ba* PTA84 and *Bs* PTA86 genomes yielded 1 contig each while the *Ba* PTA85 assembly contained 2 contigs – a large 4,084,681 bp contig and a smaller 231,132 bp long contig. The genome properties and annotation of different features are summarized in Table 1. The whole-genome sequences were deposited at DDBJ/ENA/GenBank under BioProject numbers PRJNA701126 and PRJNA701127.

**TABLE 1:** Genome assembly and annotation summary of *Bacillus* spp.

Feature	PTA-84	PTA-85	PTA-86
No. sequences	1	2	1
Total genome size (bp)	4,090,715	4,315,813	4,089,676
CDS	3,957	4,277	4,027
Miscellaneous feature	8	12	8
Mobile Elements	2	2	2
Non-coding RNA	11	11	11
Operons	753	844	747
Ribosomal RNA	27	27	30
Ribosomal binding sites	4,004	4,212	4,026
Transcription terminators	2,082	2,197	2,196
Riboswitch	44	45	48
Transfer RNA	86	87	86
Transfer-messenger RNA	1	1	1

**Genbank submission**			

BioProject	PRJNA701126	PRJNA701126	PRJNA701127
BioSample	SAMN17848991	SAMN17848992	SAMN17848993
Accession Number	CP071042	JAFMSQ000000000	CP071043

### Core-Genomes of *Ba* PTA84, *Ba* PTA85, and *Bs* PTA86

**FIGURE 4:**
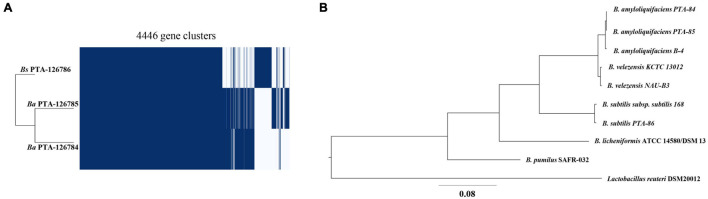
Phylogenetic analysis of poultry origin *Bacillus* spp. **(A)** Core genome analysis of *Ba* PTA84 and PTA85, and *Bs* PTA86. **(B)** Phylogenetic relationship of *Ba* PTA84 and PTA85, and *Bs* PTA86 to other *Bacillus* species along with *Lactobacillus reuteri* as an outgroup.

Ortholog analysis was performed to identify paralogous and/or orthologous relationships between the genomes of *Ba* PTA84, *Ba* PTA85, and *Bs* PTA86. *Ba* PTA84 shared the highest number of genes with 99.4% gene presentation in orthogroups while *Ba* PTA85, and *Bs* PTA86 shared 93.6% and 90.1%, respectively (Supplementary Table 8). A total of 3,024 orthologs were shared among all three strains while 586 orthologs were shared only between *Ba* PTA84 and *Ba* PTA85, 60 orthologs between *Ba* PTA84 and *Bs* PTA86, and 34 orthologs were shared only between *Ba* PTA85, and *Bs* PTA86 (Figures 4A,B).

### Phylogenetic Analysis of *Ba* PTA84, *Ba* PTA85, and *Bs* PTA86

Phylogenetic relationships of the three genomes were explored with UBCG v3.0 which employs a set of 92 single-copy core genes commonly present in all bacterial genomes. *Ba* PTA84, *Ba* PTA85 and *Bs* PTA86 genomes were compared against the genomes of *B. amyloliquefaciens*, *B. velezensis*, and *B. subtilis* strains along with *Lactobacillus reuteri* as an outgroup (Accession numbers: AL009126, CP000560, CP002627, CP002634, CP002927, HE617159, HG514499, JMEF01000001, CP005997, CP009748, CP009749, CP011115, LHCC01000001, CP014471, and QVMX01000001). As shown in Figure 4, both strains *Ba* PTA84 and *Ba* PTA85 showed closest relationship to *B. amyloliquefaciens* B4 while *Bs* PTA86 showed closest relationship to *B. subtilis* subsp. *subtilis* 168.

### Genome Analyses of *Ba* PTA84, *Ba* PTA85, and *Bs* PTA86

The assembled genome sequences of 3 *Bacillus* strains were annotated for the following potential probiotic properties, enzymes, antioxidants, bacteriocins, and secondary metabolites, and for the presence of genes of potential safety concerns such as genes encoding toxins, virulence factors, and antimicrobial resistance genes. A detailed description of each of the above-mentioned features is described below.

#### Selected Enzymes Analyses

**FIGURE 5:**
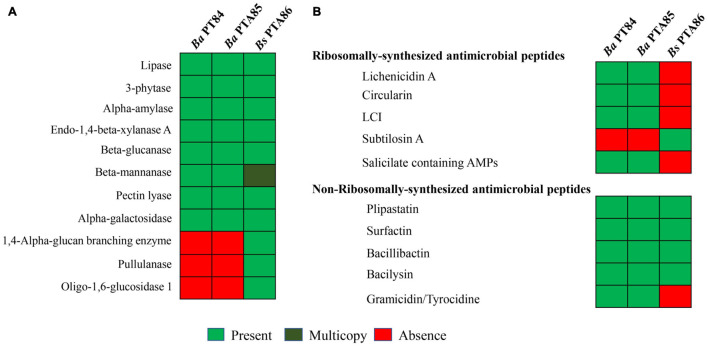
*In silico* analysis of genes encoding enzymes and antimicrobial peptides in *Bacillus* spp. genomes. Genes encoding enzyme **(A)** and antimicrobial peptides **(B)** were compared among the genomes of three *Bacillus* spp., *Ba* PTA84 and PTA85, and *Bs* PTA86.

Figure 5 illustrates the presence and absence of genes encoding selected digestive enzymes identified in the *Bacillus* genomes. All three *Bacillus* genomes encode lipase, 3-phytase, alpha-amylase, endo-1,4-β-xylanase A, β-glucanase, β-glucanase, β-mannanase, pectin lyase, and alpha galactosidase. *Bs* PTA86 carried two copies of β-mannanase genes, Supplementary Table 9. β-mannanase catalyzes the hydrolysis of β-1,4-linkage of glucomannan releasing mannan oligosaccharide (McCleary, 1978; Saeed et al., 2019). This enzyme along with phytase, xylanase, amylase are added as feed ingredients to improve feed digestibility (Fayek et al., 1995; Sherif, 2009; Zhou et al., 2009). Of the three *Bacillus* genomes, only *Bs* PTA86 possessed pullulanase, oligo-1,6-glucosidase, and glycogen degrading enzymes such as 1,4-alpha-glucan branching enzyme. A complete list of enzymes in the three *Bacillus* genomes were presented in Supplementary Table 9.

#### Secondary Metabolites

Secondary metabolite clusters accounted for 20, 20, and 12% of the genomes of *Bacillus Ba* PTA84, *Ba* PTA85, and *Bs* PTA86, respectively. Table 2 illustrates the respective clusters for each *Bacillus* genome. *Ba* PTA84 and *Ba* PTA85 genomes contained 13 secondary metabolite clusters whereas *Bs* PTA86 genome encoded for 10 clusters. More than half of the clusters were contributed by biosynthetic genes for antimicrobial peptides (AMPs) (Figure 5B).

**TABLE 2:** Secondary metabolites gene clusters of *Ba* PTA84, *Ba* PTA85, and *Bs* PTA86.

Type class/cluster*	PTA84	PTA85	PTA86
**Bacteriocin and RiPPs class**			
Amylocyclicin	1	1	0
ComX1	1	1	0
LCI	1	1	0
Lanthipeptide_class_II	1	1	0
Competence	0	0	1
Subtilosin_(SboX)	0	0	1

**Secondary metabolite biosynthesis gene cluster**			

CDPS	0	0	1
NRPS	1	1	2
NRPS, PKS-like, T3PKS, transAT-PKS, transAT-PKS-like	0	0	1
NRPS, T3PKS, transAT-PKS, transAT-PKS-like	1	1	0
NRPS, bacteriocin	1	1	0
NRPS, beta-lactone	0	0	1
NRPS, beta-lactone, transAT-PKS	1	1	0
NRPS, transAT-PKS	1	1	0
PKS-like	1	1	0
T3PKS	1	1	1
Head_to_tail, sactipeptide	0	0	1
Lanthipeptide	1	1	0
Terpene	2	2	2
transAT-PKS	1	1	0
transAT-PKS, transAT-PKS-like	1	1	0
Other	1	1	1

*RiPP, ribosomally synthesized and post-translationally modified peptides; NRPS, non-ribosomal peptide synthase; PKS, polyketide synthase; T3PKS, type III polyketide synthase; *trans*-Type 3-PKS; AT-PKS, *trans*-acyltransferase polyketide synthase.

#### Genes of Safety Concern

To search for genes encoding known virulence factors, toxins, and antimicrobial resistance (AMR), we applied a screening approach using cutoff values according to an EFSA guideline (EFSA, 2021), sequence identity and coverage values higher than 80 and 70%, respectively. According to the analysis, genes for known virulence factors or toxins were not identified in the genomes of three *Bacillus* strains, *Ba* PTA84, *Ba* PTA85, and *Bs* PTA86. Supplementary Table 10 presents genes for putative genes encoding for antimicrobial resistance (AMR). *Ba* PTA84 and 85 had a similar set of putative AMR genes identified, namely putative genes encoding methyl transferase (*cfr/cfr*-like, *clb*A) (EFSA Panel on Additives and Products or Substances used in Animal Feed [FEEDAP] et al., 2018), tetracyclin efflux protein [*tet*(L)] (World, 2021), Streptothricin-*N*-acetyltransferase (*sat*A), and rifamycin-inactivating phosphotransferase (*rph*C) (Laslett and Canback, 2004; Hyatt et al., 2010). *Bs* PTA86 genome carried putative genes that encoded macrolide 2′phosphotransferase (*mph*K), ABC-F type ribosomal protection protein (*vml*R), Streptothricin-*N*-acetyltransferase (*sat*A), tetracyclin efflux protein [*tet*(L)], aminoglycoside 6-adenylyltransferase (*aad*K) (Seemann, 2014), and rifamycin-inactivating phosphotransferase (*rph*C). The *aad*K gene from *B. subtilis* was originally found in susceptible derivatives of Marburg 168 strains. Heterologous expression of the gene in a plasmid in *E. coli* resulted in resistance phenotype toward rifamycin suggesting the need for high gene copies to confer resistance (Suzek et al., 2001).

#### Antioxidants, Adhesion, and Folate Biosynthesis

Genes encoding primary redox enzymes such as superoxide dismutase and catalase that scavenge reactive oxygen species were found in the three *Bacillus* genomes, Supplementary Table 11. A thioredoxin system and genes for bacillithiol biosynthesis were also identified. All three genomes encoded for a thioredoxin reductase (locus tag for *Ba* PTA84, *Ba* PTA85, and *Bs* PTA86: JS608_03853, JTE87_00428, and JS609_03503, respectively) and two cognate thioredoxins for *Ba* PTA84 and *Ba* PTA85 (locus tags, JS608_02520 and JTE87_01059; JS609_02844 and JS608_03225) and a Trx for *Bs* PTA86 (locus tag, JTE87_01767). Thioredoxin systems maintain cellular redox homeostasis (Meyer et al., 2009). Interestingly, despite lacking glutathione-glutaredoxin system, several genes for glutathione transport were found suggesting the potential transport of redox proteins, possibly bacillithiol, to the extracellular environment maintaining redox potential of the surroundings. Two genes for bacillithiol biosynthesis (Gaballa et al., 2010), *bsh*A and B, were identified in genomes of *Ba* PTA84 and PTA85, and *Bs* PTA86, Supplementary Table 11.

Two genes each encoding for elongation factor Tu and 60 kDa chaperonin involved in adhesion of *Bacillus* species to intestinal epithelium were identified in all three genomes.

Probiotic bacteria confer several health benefits to the host, including vitamin production. We searched for key components of folate production pathways in *Bacillus* strains using the Enzyme Commission (EC) numbers associated with folate biosynthetic pathway. The analysis of genome sequences of *Bacillus* strains identified genes involved para-aminobenzoic acid (PABA) synthesis in all three strains (Table 3). However, strain *Ba* PTA84 has a frameshift mutation in *pab*B gene. The enzymes necessary for chorismate conversion into PABA are present in all three *Bacillus* probiotic strains. *Bacillus* probiotic strains also contain the genes of DHPPP *de novo* biosynthetic pathway. Previous studies have shown that *B. subtilis* genome harbor all the pathways components and have been engineered for folate production (Salem et al., 1972; Zhu et al., 2005; de Crecy-Lagard, 2014).

**TABLE 3:** Genes involved in folate biosynthetic pathway in probiotic *Bacillus* spp.

Annotation	Gene	EC number	PTA-84	PTA-85	PTA-86
Protein AroA(G)	*aro*A	5.4.99.5	JS608_03349	JTE87_00934	JS609_02973
3-phosphoshikimate 1-carboxyvinyltransferase 1	*aro*A1	2.5.1.19	JS608_02738	JTE87_01546	JS609_02206
3-dehydroquinate synthase	*aro*B	4.2.3.4	JS608_02748	JTE87_01536	JS609_02216
Chorismate synthase	*aro*C	4.2.3.5	JS608_02749	JTE87_01535	JS609_02217
3-dehydroquinate dehydratase	*aro*D	4.2.1.10	JS608_01242	JTE87_03044	JS609_02255
Shikimate dehydrogenase [NADP(+)]	*aro*E	1.1.1.25	–	–	JS609_02520
Shikimate dehydrogenase [NADP(+)]	*aro*E_1	1.1.1.25	JS608_01241	JTE87_01221	–
Shikimate dehydrogenase [NADP(+)]	*aro*E_2	1.1.1.25	JS608_03061	JTE87_03045	–
Chorismate mutase AroH	*aro*H	5.4.99.5	JS608_02747	JTE87_01537	JS609_02215
Shikimate kinase	*aro*K	2.7.1.71	JS608_00741	JTE87_03543	JS609_00356
Aromatic amino acid transport protein AroP	*aro*P	–	–	–	JS609_00352
Dihydroneopterin aldolase	*fol*B	4.1.2.25	JS608_00496	JTE87_03790	JS609_00092
Bifunctional protein FolD protein	*fol*D	1.5.1.5	JS608_02930	JTE87_01353	JS609_02382
GTP cyclohydrolase 1	*fol*E	3.5.4.16	JS608_02756	JTE87_01528	JS609_02224
GTP cyclohydrolase FolE2	*fol*E2	3.5.4.16	–	–	JS609_00375
2-amino-4-hydroxy-6- hydroxymethyldihydropteridine pyrophosphokinase	*fol*K	2.7.6.3	JS608_00497	JTE87_03789	JS609_00093
Dihydropteroate synthase	*fol*P	2.5.1.15	JS608_00495	JTE87_03792	JS609_00091
Dihydropteroate synthase	*fol*P1	2.5.1.15	–	JTE87_03791	–
Aminodeoxychorismate/anthranilate synthase component 2	*pab*A	2.6.1.85	JS608_00493	JTE87_03794	JS609_00089
Aminodeoxychorismate synthase component 1	*pab*B	2.6.1.85	JS608_00492*	JTE87_03795	JS609_00088
Alkaline phosphatase D	*pho*D	3.1.3.1	JS608_00693	JTE87_03591	JS609_00303
Alkaline phosphatase 3	*pho*B	3.1.3.2	–	–	JS609_00619
Alkaline phosphatase 4	*pho*A	3.1.3.3	JS608_01407	JTE87_02880	JS609_01001
Dihydrofolate reductase type 3	*dhfr*III	1.5.1.3	JS608_02513	JTE87_01774	–
					

*The asterisk means pseudogene.*

### Screening for Prophages, Insertion Sequences and Transposases

All three strains were scanned for the presence of mobile genetic elements such as prophages, insertion sequences (IS) and transposases. Both *Ba* PTA84 and *Ba* PTA85 have 3 transposases while *Bs* PTA86 has 4 transposases. *Ba* PTA84, *Ba* PTA85 and *Bs* PTA86 share 2 copies of IS21 insertion sequence.

### Effects of In-Feed Administration of *Ba* PTA84 on Growth Performance of Broiler Chickens

**FIGURE 6:**
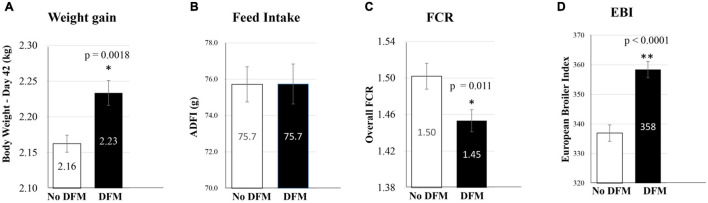
Effect of in-feed administration of *Ba* PTA-84 on growth performance of broiler chicken. Growth performance as measured by four parameters, **(A)** weight gain, **(B)** feed intake, **(C)** Feed Conversion Ratio (FCR), and **(D)** European Broiler Index (EBI).

As a proof of principle, *Ba* PTA84 was selected in an *in vivo* pilot study as a DFM to evaluate its probiotic efficacy in supporting improved broiler growth performance. To accomplish this, 2,500 1-day old broiler chickens (Cobb 500) were randomly assigned to 50 pens of 50 birds each and split between two treatment groups; an untreated Control group and a group receiving 1.5 × 10^5^ CFU of *Ba PTA*84 per gram of feed for the full 42-day production period. Despite similar feed intake, birds fed the DFM had 3.5% higher final body weight compared to the control (2.16 vs. 2.23 kg for Control and DFM, respectively; *p* = 0.0018). This translated to a 3.3% improvement in feed conversion ratio (FCR) for the DFM-fed group compared to Control (1.50 vs. 1.45 for Control and DFM, respectively; *p* = 0.011) and a 6.2% increase in the European Broiler Index (EBI), a metric of overall production efficiency (337 vs. 358 for Control and DFM, respectively; *p* < 0.0001), Figure 6. These results indicated that use of *Ba* PTA84 as a DFM can significantly improve weight gain and feed efficiency in broiler chickens.

### Cecal Microbiome Structure Analysis From Chicken With and Without In-Feed Administration of *Ba* PTA84

To gather insights into the *in vivo* effects of in-feed administration of *B. amyloliquefaciens* on broiler chickens, we analyzed the cecal microbiomes of animals in the control and treatment groups of our clinical study. Samples from twenty 42-day old animals from each treatment group were used to build 16S rRNA amplicon libraries for sequencing, corresponding to one randomly chosen animal per pen. The median coverage was ∼36,000 read pairs per sample, and 1,945 ASVs were identified across samples.

**FIGURE 7:**
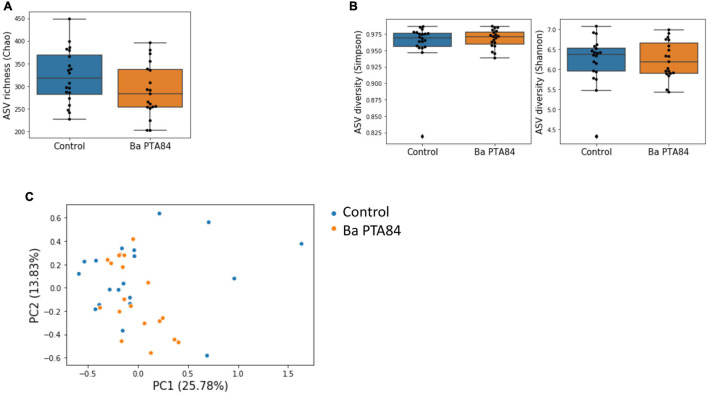
Microbiome analysis of cecal content from birds fed with or without *Ba* PTA84. **(A)** ASV richness in the cecum of control birds and birds treated with *Ba* PTA84. Richness is quantified using the Chao index (Mann–Whitney *U p*-value = 0.07). **(B)** ASV diversity quantified with the Simpson **(left)** and Shannon indexes **(right)** for control birds and birds treated with *Ba* PTA84. (*p*-value = 0.44, 0.36). **(C)** Principal component analysis of the Bray–Curtis dissimilarity matrix between microbiome profiles for control birds and birds fed with *Ba* PTA84. Each dot represents a cecal sample. Numbers in parentheses indicate the variance explained by each principal component.

Samples in the control and treatment groups showed similar values for ASV richness and diversity (Figures 7A,B, *p*-values = 0.07, 0.44–0.36, respectively). Additionally, ANOSIM and PERMANOVA analyses based on the Bray–Curtis dissimilarity between samples did not support significant differences in community structures between treatment groups (ANOSIM *p*-value = 0.66. PERMANOVA *p*-value = 0.44). In addition, using ANCOM, we did not observe differentially abundant ASVs between treatment groups, including those classified to the genera of pathogens used for *in vitro* screening (Mandal et al., 2015). These results, which are evident by the lack of clustering of samples by treatment following principal components analysis (Figure 7C), indicate that supplementation of *Ba* PTA84 at a dose sufficient to induce a positive effect on performance (Figure 7) had minor effects on the diversity and structure of the cecal microbiome.

## Discussion

A clear understanding of the physiology and safety of probiotic strains as well as their interactions with target host, and hosts’ gut microbiota are essential to rationally develop the next generation of probiotics with improved safety and efficacy, and increased reproducibility. Here, we employed comprehensive multi-omics, biochemical, and microbiological approaches for the selection and characterization of *Bacillus* spp. strains to improve growth performance in poultry. Our data showed that the selected strain *Ba* PTA84 significantly improved growth performance indicating that the screening workflow helped to rationally design promising DFM candidates. Moreover, we generated copious data that we expect will guide future efforts to decipher the gene clusters, metabolites, phenotypic traits, and microbiome impact of spore-formers as important characteristics of probiotic strains.

**FIGURE 8:**
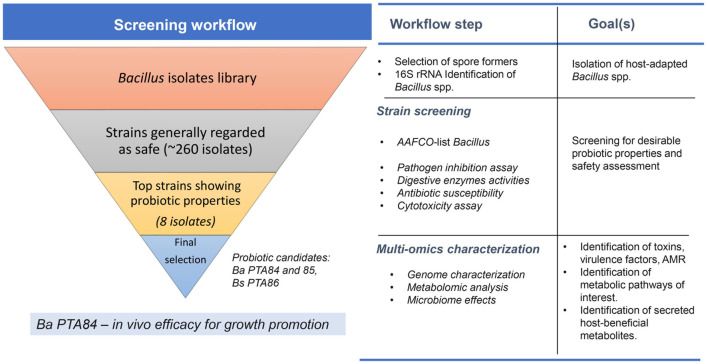
A screening workflow diagram for selection of *Bacillus* spp. probiotic candidate. *Bacillus* spp. isolates were screened for their activities to inhibit poultry pathogens and ability to secrete digestive enzymes *in vitro*. The best candidates were further selected based on their safety profiles (i.e., antimicrobial resistance profile and cytotoxicity level). Genomic and metabolomic analyses were performed on the select isolates to further investigate potential host-benefit properties and possible health/safety concerns. The top candidate was then tested for its effects on growth promotion *in vivo*.

Figure 8 illustrates the screening and characterization workflow. This bottom-up approach ensures selection of the best candidates at each screening step. Strains that did not meet safety criteria were not selected. Only the best candidates that met phenotypic selection criteria moved forward to the next screening step. Genomic analysis of the top three *Bacillus* strains helped to create a link between phenotypic observations with genomic traits. Furthermore, genomic and metabolomic analyses of three candidate strains pointed to potential outcome differences when combining these three candidate strains in a consortium. Details from our findings are described below.

### Host-Adapted *Bacillus* Strains

We expected that host-adapted *Bacillus* strains to exert better probiotic effects in the host environment than those isolated from other sources, thus, we targeted our isolation to those *Bacillus* spp. from animal GIT content or fecal samples of healthy animals (Barbosa et al., 2005). A higher diversity of isolates was obtained from the ethanol-treated samples compared to heat-treated samples, as reported previously (Koransky et al., 1978; Barbosa et al., 2005). Despite the general heat resistance feature of *Bacillus* spores, spore core, cortex, coat, and membrane composition determines the degree of the spores’ heat resistance (Setlow, 2006; Brul et al., 2011; Berendsen et al., 2015) resulting in different responses of spores toward heat stresses.

### Desirable Probiotic Properties

With the continuing reduction in use of antibiotics in poultry farms, as driven by regulations, and some customer preferences, the development of microbial feed additives that support maintenance of poultry health in the face of undesirable organisms would be beneficial. Our screening results showed that *Bacillus* spp. controlled the growth of undesirable *E. coli* O2, O18, and O78, *C. perfringens*- and *Salmonella* Typhimurium. APEC strains cause colibacillosis, which is a major problem in commercial production (Dziva and Stevens, 2008; Mellata, 2013). Colibacillosis occurs when APEC originating from fecal materials translocate into the lung epithelium during fecal aerosolization. Thus, reducing the APEC load in feces as a potential effect of *Bacillus* spp. in the feed could help reduce the incidence of colibacillosis (Duan et al., 2008; Chien et al., 2011). *C. perfringens* is a pathogen that causes necrotic enteritis in poultry (Cooper and Songer, 2009) by the production of alpha toxin and NetB (Keyburn et al., 2006, 2008). Necrotic enteritis is a multi-factorial disease that cost poultry farmers 6 billion dollar annually (Wade and Keyburn, 2015). *Salmonella* Typhimurium, a poultry gut commensal, is the major cause of salmonellosis in humans. This infection is facilitated by the consumption of *Salmonella*-containing poultry products (Foley and Lynne, 2008; Batz et al., 2012). The ability of *Bacillus* spp. to suppress the growth of these undesirable organisms might be due to the production of AMPs (bacteriocins). Genome analysis of *Ba* PTA84, *Ba* PTA85, and *Bs* PTA86 suggested that *Ba* and *Bs* genomes encoded distinct AMPs (Figure 5B). *Ba* PTA84 and *Ba* PTA85 genomes encoded for ribosomally-synthesized lichenicidin A, circularin, LCI, and salicylate containing AMPs that were not found in *Bs* PTA86 genome. The latter possessed subtilosin A, a cyclic antimicrobial peptide that are potent against some Gram-positive and Gram-negative bacteria such as *Listeria monocytogenes, Enterococcus faecalis, Porphyromonas gingivalis, Klebsiella rhizophila, Streptococcus pyogenes* and *Shigella sonnei*, *Pseudomonas aeruginosa* and *Staphylococcus aureus* (Babasaki et al., 1985; Stein et al., 2004; Shelburne et al., 2007). For non-ribosomally synthesized AMPs, *Ba* PTA84 and *Ba* PTA85 carried plipastatin, surfactin, bacillibactin, bacilysin, and gramicidin. Only the latter was absent in *Bs* PTA 86.

*Bacillus* species are known to secrete host beneficial enzymes such as cellulase, xylanase, amylase, protease, β-mannanase, phytase (Latorre et al., 2016; Singh and Bajaj, 2016; Danilova and Sharipova, 2020). These enzymes, when fed to animals, improve digestion of low-calorie diets or reduce intestinal inflammation by breaking down non-starch polysaccharides (NSPs). Some NSPs are anti-nutritional factors, and increase the gut content viscosity, slow down feed retention time in the gut, and thus reduce nutrient absorption (Raza et al., 2019). An accumulation of undigested NSPs can lead to the growth of pathogens that cause subclinical infection challenges (Kaldhusdal, 2000; Van Immerseel et al., 2004). Production of pro-inflammatory cytokines as a response to NSPs demands a significant amount of energy, which otherwise could be preserved for growth, lowering food efficiency and growth performance [reviewed in Cardoso Dal Pont et al. (2020)]. *Ba* and *Bs* showed comparable protease, amylase, and β-mannanase activities. These activities were supported by our genomic analysis showing that *Ba* and *Bs* possess genes encoding for amylase, protease, β-mannanase, and phytase. *Bs* PTA86 genome contained more genes for enzymes compared to those of *Ba* PTA84 and PTA 85.

It is noteworthy that genome analyses revealed other potential benefits of top three *Bacillus* candidates for animals. Genes encoding a wide array of antioxidant proteins were identified, superoxide dismutase, catalase, thioredoxin, and methionine sulfoxide, and bacillithiol. These proteins when expressed and secreted in the GIT could provide protection toward oxidative stress (Abudabos et al., 2016; Wang et al., 2017; Inatomi and Otomaru, 2018). Oxidative stress occurs in the GIT when the level of free radicals generated by reactive oxygen/nitrogen species (RO/NS) is much higher than the level of antioxidant proteins for neutralization of these toxic compounds (Sherif, 2009). This event is triggered by various factors including nutritional or environmental heat stress, or pathological factors which ultimately decrease growth performance and quality of meat and eggs (Sherif, 2009). Another key desirable traits in a probiotic candidate is the ability to adhere to epithelial cells. The two genes identified in all three strains encode proteins potentially involved in adhesion to mucus, epithelial cells and are known to be involved in host immunomodulation and unwanted microorganism aggregation, providing stability to the strains and the ability to compete with other undesirable resident gut bacteria, thereby enabling effective colonization of the gut and exclusion of pathogens (Antelmann et al., 2000; Sánchez et al., 2008).

Among proposed functions of probiotic bacteria are the reduction of potential pathogenic bacteria, immune modulation, removal of harmful metabolites in the intestine and/or providing bioactive or otherwise regulatory metabolites. Folate-producing probiotic bacteria enable better nutrient digestion and energy recovery. Folate-producing probiotic strains could potentially confer protection against cancer, inflammation, stress, and digestive disturbances (White et al., 1997; Fuchs et al., 2002; Zhu et al., 2005; Rossi et al., 2011; Zhang et al., 2020). Several studies exploring the commercial utility of probiotic strains for folate production have been reported (Sauer et al., 1998; Schallmey et al., 2004; Rossi et al., 2011). Genes encoding essential enzymes in the biosynthetic pathways of folate were also found in the genome of three *Bacillus* strains. The products of these pathways supply important cofactors which once secreted would be absorbed by the host improving health status (Sauer et al., 1998; Schallmey et al., 2004; Rossi et al., 2011).

### Safety Profiles

In addition, *Bacillus* DFM candidates must have acceptable safety profiles as expected by regulatory authorities. Some *Bacillus* spp. are known to produce AMPs and enterotoxins that might exert deleterious effects on the host cells (EFSA Panel on Additives and Products or Substances used in Animal Feed [FEEDAP] et al., 2018). Lack of detailed characterizations of probiotic strains resulted in the use of *B. cereus* (Bactisubtil, Biosubtyl, and Subtyl) probiotic strains harboring structural genes of known enterotoxins (Duc et al., 2004). Cytotoxicity assessment of our *Bacillus* spp. strains suggested that *Bacillus* spp. did not cause cytotoxicity of Vero cells. Moreover, genome analysis of *Ba* PTA84, *Ba* PTA85, and *Bs* PTA86 suggested that enterotoxins and other known virulence factors were absent in the subject *Bacillus* spp. Another important safety criterion is that *Bacillus* DFM genomes must be devoid of transferable antimicrobial resistance genotypes (Ciorba et al., 2015). The data suggested that almost all of the tested *Bacillus* isolates were sensitive to the antimicrobials tested and the apparent MIC values were below the recommended cut-off values. Genomic analysis of three *Bacillus* spp. identified putative genes for antimicrobial resistance to tetracycline, lincosamide, and streptothricin. In the genome of *Bs* PTA86, putative genes conferring resistance to rifampicin and macrolides were found. However, these genes have been reported present in *Ba* and *Bs* isolates from the environment (Phelan et al., 2011; Adimpong et al., 2012), suggesting these genes may be intrinsic properties of *Ba* and *Bs* strains. Furthermore, transferable mobile genetic elements such as transposons, insertion sequences were absent in the proximity of these genes indicating the very low risk of these genes being horizontally transferred to other gut microbes pose little to no risk to public health safety.

### Metabolomic Analysis

To investigate the potential host beneficial metabolites secreted by the three *Bacillus* strains, we performed global untargeted metabolomic analyses of *Ba* PTA84 and PTA85, and *Bs* PTA86. The use of rich and minimal media, as well as the co-culture experiments, allowed us to identify a collection of global secreted molecules of potential value. A metabolite of particular interest was 1-kestose that was identified in the culture supernatants of all strains. 1-Kestose, the smallest fructooligosaccharide (FOS), is a trisaccharide molecule composed of a glucose and two fructose residues linked by glycosidic bonds. Kestose is a prebiotic that, when consumed, enriches the growth of gut commensals such as Bifidobacteria, Lactobacillus, and *Faecalibacterium prausnitzii* promoting gut health (Patterson et al., 1997). Of the three strains, *Ba* PTA84 produced the highest amount of 1-kestose. Thioproline, an antioxidant molecule, was identified in the culture supernatant of *Ba* PTA84 and *Bs* PTA86. Thioproline was reported to inhibit carcinogenesis in humans, and is expected to act as a nitrite scavenger (Patterson et al., 2020). Pantothenate (Vitamin B5) and pyridoxine (Vitamin B6) were found in the culture supernatants of *Ba* PTA85 and *Bs* PTA86, respectively. Betaine and choline were possibly secreted by *Bs* PTA86. These molecules are methyl donors required for the biosynthesis of acetylcholine and phosphatidylcholine, for neural transmission and cell membrane integrity, respectively (Zempleni et al., 2007). Betaine, when supplemented in feed, has shown improved growth performance of birds during heat stresses (He et al., 2015; Liu et al., 2019). Inclusion of choline has been associated with reduced FCR in broiler chickens (Santiago et al., 2020).

Genomic analysis of *Ba* PTA84, PTA85, and *Bs* PTA86 suggested that these three strains harbor genes with complimentary activities (i.e., AMPs and enzymes). Thus, we hypothesize that inclusion of these three strains in a consortium would exert a greater benefit to the animal than that of any single strain. It is noteworthy that the subject *Bacillus* spp. generated more unique metabolites when grown in consortia of two or three *Bacillus* strains, suggesting that a combination of strains would generate distinct outcomes compared to that of single isolate (Figure 5). Indoleacetate was detected only in the culture supernatant of consortia of *Ba* PTA84-*Ba* PTA85, and *Ba* PTA84-*Ba* PTA85-*Bs* PTA86, but not in the individual strains. Indolacetate is an intermediate of microbial tryptophan biosynthesis that serves as a ligand for aryl hydrocarbon receptors (AhRs) enhances intestinal integrity and modulates host immune systems by exerting anti-inflammatory activities (Nawrocki et al., 2009; Tjaden, 2020). A higher abundance of tryptophan metabolites was also observed in animals treated with sub-therapeutic level of antibiotic Bacitracin Methylene Disalicylate (BMD) (Xie and Tang, 2017).

### Feed Inclusion of *Ba* PTA84 Supported Improved Poultry Growth Performance

An *in vivo* efficacy study employing daily feed inclusion of *Ba* PTA84 resulted in significantly improved overall growth performance of broiler chickens as shown by a significant increase of ADG, production efficiency, and a reduction in feed conversion ratio. To better understand the mechanism of action underlying the effect of *Ba* PTA84 supplementation on animal health, we investigated the effects of supplementation of *Ba* PTA84 on the modulation of intestinal microbiota. The chicken gut microbiota plays prominent roles in bioavailability of nutrients, immune system development, intestinal integrity, and exclusion of unwanted microorganisms (Shang et al., 2018). Growth promotion effects of probiotics have been linked to changes in cecal microbiome structure and function (Al-Fataftah and Abdelqader, 2014; Ma et al., 2018). Interestingly, microbiome taxonomic profiling analyses of cecal contents from a control group and that with dietary supplement of *Ba* PTA84 suggested no significant differences between the cecal microbiome structures of the two groups according to both alpha and β-diversity parameters. Thus, it is likely that *Ba* PTA84 supports growth performance without altering the normal cecum microbiome. It is still possible that microbial communities in other organs are affected by supplementation of this strain. It is noteworthy that metabolomic analysis of culture supernatant of *Ba* PTA84 showed that it had a potential to produce 1-kestose, a microbiome modulator (Patterson et al., 1997). In the future, it would be interesting to test whether 1-kestose is indeed produced *in vivo*.

## Conclusion

*Ba* PTA84 was selected via a comprehensive screening workflow as a potential probiotic for supporting improvement of growth performance in broiler chickens. Inclusion of *Ba* PTA84 in one study resulted in significantly improved weight gain and overall animal growth performance. More detailed studies on the mechanisms of action of *Ba* PTA84 are yet to be done. This includes investigating the effects of *Ba* PTA84 on intestinal structure, immune system function, oxidative response, microorganism exclusions, and nutrient digestibility. It is interesting to see in the future whether in-feed inclusion of other well-characterized probiotic candidates such as *Ba* PTA85 and *Bs* PTA86 or multi-strain consortia harboring a combination of genes and traits will have better outcomes than that of *Ba* PTA84. With the advances of sequencing technologies allowing analysis of large number of samples with relatively low cost, it is possible to use genomic analysis as an initial high-throughput screening step to eliminate candidate strains harboring genes that might have negative impacts to the animal or to public health, and importantly to enable investigating the impact of individual genes and molecules on the observed clinical outcomes.

## Data Availability Statement

The raw sequencing reads, genome assemblies and annotations in this study were deposited in the NCBI BioProject database under projects PRJNA701126 and PRJNA701127. The accession numbers of CP071042, JAFMSQ000000000, and CP071043 were assigned to *B. amyloliquefaciens* ATCC PTA-126784 and ATCC PTA-126785, and *B. subtilis* ATCC PTA787 126786, respectively. Raw reads for the 16S rRNA cecal microbiome analysis were submitted to the NCBI Sequence Read Archive with accession number: PRJNA721687.

## Ethics Statement

The animal study was reviewed and approved by Elanco Institutional Animal Care and Use Committee (Protocol #1395).

## Author Contributions

DS, NB, DG, TH, SPM, and AK conceived and designed the experiments. DS, AV, NB, and DG performed the experiments. DS, AV, NT, NB, DG, GP, AN, SPM, and AK analyzed and interpreted data. DS, AV, NB, GP, and SPM wrote the manuscript. All authors read, reviewed, and approved the final manuscript.

## Conflict of Interest

All authors are employees of Elanco Animal Health. Elanco Animal Health manufactures and markets probiotics.

## Publisher’s Note

All claims expressed in this article are solely those of the authors and do not necessarily represent those of their affiliated organizations, or those of the publisher, the editors and the reviewers. Any product that may be evaluated in this article, or claim that may be made by its manufacturer, is not guaranteed or endorsed by the publisher.

## Footnotes

1
http://scikit-bio.org/

## Abbreviations

DFM: direct fed microbials
Ba: *Bacillus amyloliquefaciens*
Bs: *Bacillus subtilis*
ADG: average daily gain
FCR: feed conversion ration
EBI: European Broiler Index
APEC: Avian Pathogenic *Escherichia coli*
MIC: minimum inhibitory concentration
EFSA: European Food Safety Association
AAFCO: Association of American Feed Control Officials
ASV: amplicon sequence variant
PCA: principal component analysis.
