# Supplementary material for: Multi-Omics Characterization of Host-Derived Bacillus spp. Probiotics for Improved Growth Performance in Poultry
Source: Front Microbiol. 2021 Oct 20;12:747845. doi: 10.3389/fmicb.2021.747845 (PMC8563996; doi:10.3389/fmicb.2021.747845)
Supplement: Supplementary file 1 [file Data_sheet_1.docx]

**SUPPLEMENTARY MATERIALS**

**Multi-omics characterization of host-derived *Bacillus* spp. probiotics for improved growth performance in poultry**

Dwi Susanti^1^*, Alyssa Volland^1^, Nilesh Tawari^2^, Nielson Baxter^3^, Dharanesh Gangaiah^1^, Germán Plata^2^, Akshitha Nagireddy^2^, Troy Hawkins^2^, Shrinivasrao P. Mane^2^, Arvind Kumar^1,^*

Discovery Biology, Bacteriology and Microbiome^1^, Global Computational Sciences^2^, and Nutritional Health^3^ Divisions, Elanco Animal Health, Greenfield, IN

***Correspondences:**

Dwi Susanti

dwi.susanti@elancoah.com

Arvind Kumar

arvind.kumar@elancoah.com

**Supplementary Methods**

***In vitro* microbial inhibition assay**

The assays were modified from a protocol described in (Latorre et al., 2016) and performed in duplicate. Briefly, 10 µl of *Bacillus* freezer stock was inoculated into 2 mL of 0.5x LB in a 15 mL round bottom shaker tube. The cultures were incubated at 37^o^C for 48 hours while shaking at 200 rpm. For APEC strains and *S.* Typhimurium, 50 µl of freezer stock was inoculated into 5 mL of LB in a 15 mL round bottom shaker tube. The cultures were incubated at 37^o^C overnight while shaking at 200 rpm. Once pathogens had grown overnight in liquid culture, 1.0 x 10^5^ cfu/ml of the overnight culture were inoculated into freshly prepared LB soft agar (0.8% w/v) that was cooled in a water bath set to 45^o^C after autoclave sterilization. 5 mL of the molten agar was aliquoted into each well of a 6-well cell culture plate (2 wells per *Bacillus* strain plus the negative control). The soft agar was solidified and air-dried for 3-4 hours. Onto this agar, 5 µl of 48-hour *Bacillus* culture were applied to the center of each well. The plates were inverted and allowed to incubate overnight at 37^o^C for 24 hours and zones of inhibition were observed and recorded.

For *Clostridium perfringens* screening, 5 mL of molten LB agar (1.5%, w/v) were aliquoted into each well of a 6-well cell culture plate and allowed to solidify overnight. Then 5 µl of 48-hour *Bacillus* culture were spotted onto the center of each well. The plates were inverted and allowed to incubate overnight aerobically at 37^o^C. A colony of *Clostridium perfringens* NAH 1314-JP1011 was inoculated in liquid BYC broth an incubated overnight at 39^o^C in the anaerobic chamber. Freshly prepared BYC soft agar (0.8%, w/v) was autoclaved and allowed to cool in a water bath set to 45^o^C. Once cooled, the overnight *C. perfringens* culture was inoculated into molten soft agar at 1.0 x 10^5^ cfu/ml and mixed on a stir plate. 5 mL of the molten agar was aliquoted on top of each well of the 6-well cell culture plates containing *Bacillus* spots. As a negative control, *C. perfringens*-containing molten agar was poured onto LB agar without *Bacillus*. Once solidified, plates were inverted and allowed to incubate anaerobically overnight at 39^o^C for 24 hours. Then, zones of inhibition were observed and recorded.

**Enzyme activities**

β-mannanase assay was adapted from a protocol as described by Cleary, B., et. al. (V.McCleary, 1978). Assays for amylase and protease followed protocols in (Latorre et al., 2016).

For testing β-mannanase activity, *Bacillus* strains were grown in 5 milliliters of LB medium in a 15 mL culture tube overnight at 37^o^C while shaking at 200 rpm. Then 5 µl of 24-hour *Bacillus* culture were spotted in duplicate onto the center of an LB agar plate containing 100 mM CaCl_2._ The agar plates were incubated overnight at 37^o^C. Fresh soft agar containing Azo-carob Galactomannan (0.5%, w/v), agar (0.7%, w/v), dissolved in 50 mM Tris-HCl pH 7.0 buffer was autoclaved and allowed to cool in a water bath set to 45^o^C. Once cooled, the soft agar substrate was overlayed on to agar plates containing *Bacillus* colonies until each colony was surrounded by substrate. The plates were incubated overnight at 37^o^C and allowed to incubate for 48 hours. The zone of clearance due to β-mannanase activity could be directly visualized and recorded.

For the amylase assay, agar plates containing the following ingredients were used (entity, g/L): Tryptone, 10, Soluble starch, 3, KH_2_PO_4_, 5, Yeast extract, 10, Noble Agar, 15. An overnight culture of *Bacillus* isolates in 0.5x LB was used as an inoculum. The *Bacillus* culture was spotted onto the above plate containing soluble starch and the inoculated plates were incubated at 37^o^C for 48 hours. The zone of clearance due to amylase activity was visualized by flooding the surface of the plates with 5 mL of Gram’s iodine solution.

For testing protease activity, agar plates containing the following ingredients were used (entity, g/L): skim milk, 25, noble agar, 25. An overnight culture of *Bacillus* isolates in 0.5x LB was used as inoculum. The *Bacillus* culture was spotted onto the above plate containing soluble starch and the inoculated plates were incubated at 37^o^C for 24 hours. The zone of clearance due to protease activity could be directly visualized.

**Cytotoxicity Assay**

*Bacillu*s spp. strains were grown in 5 mL Brain Heart Infusion (BHI) liquid medium at 30^o^C overnight. This overnight culture served as an inoculum for 5 mL fresh LB, the inoculated medium was then incubated at 30^o^C for 6 hours without shaking. The expected cell density was at least 10^8^ CFU/mL. The culture was then centrifuged at 1,700 xg for 1 hour to generate cell-free culture supernatant.

200 µL serum-free medium were added to the 100% confluent Vero cells grown on 96-well plates generated following the protocol described in Materials and Methods. The cells were then exposed to 100 µL of cell-free culture supernatant of *Bacillus* spp. and the mixture was incubated inside a CO_2_ incubator (5% v/v headspace of CO_2_, Thermo Scientific, Waltham, MA) at 37 °C for 3 hour. The corresponding cell-culture medium was used in the control wells. *B. cereus* and *B. licheniformis* were used as positive and negative controls, respectively, and 0.1% Triton-X, 100 µL was used as a positive cytotoxicity control. The assay was performed in three technical replicates with three biological replicates.

At the end of the incubation period, culture supernatants were collected by centrifugation at 300 xg for 5 min. Culture supernatants from technical replicate wells were combined. Four micro liters of the culture supernatant were used for a lactate dehydrogenase assay (Sigma Aldrich,St. Louis, MO) with a total volume of 100 µL, following the protocol as described in (EFSA Panel on Additives and Products or Substances used in Animal Feed (FEEDAP), 2018). The reaction was monitored at an absorbance of 450 nm at 37^o^C for 10 minutes measuring the generation of NADH from NAD^+^ as products from lactate dehydrogenase reaction. The percent cytotoxicity level was calculated by the following formula.

% Cytotoxicity = (A_450nm__sample – A_450nm__cell-culture media control)

(A_450nm__Triton X – A_450nm__cell-culture media control)

The A_450nm_ value is an average of three biological replicates. A cytotoxicity percentage value higher than 20 was considered cytotoxic. The assays were repeated if cytotoxicity percentage of *B. cereus*, a positive control, was less than 40 or that of *B. licheniformis*, a negative control, was higher than 20.

**Global untargeted metabolomic analysis**

Metabolite analysis was performed at Metabolon, Inc. utilizing non-targeted UPLC-MS/MS approach employing a Waters ACQUITY ultra-performance liquid chromatography (Waters, Milford, MA) and a Q-Extractive high resolution/accurate mass spectrometer (Thermo Scientific, Waltham, MA) interfaced with a heated electrospray ionization (HESI-II) source and Orbitrap mass analyzer operated at 35,000 mass resolution. The samples were dried, reconstituted and aliquoted into four samples for the following analyses, a) Analysis of hydrophilic compounds employing acidic positive ion conditions with a C18 column (Waters UPLC BEH C18-2.1x100 mm, 1.7 µm) using water and methanol, containing 0.05% perfluoro pentanoic acid (PFPA) and 0.1% formic acid (FA), b) Analysis of more hydrophobic compounds employing a similar system as mentioned above except the mobile phase used was methanol, acetonitrile, water, 0.05% PFPA and 0.01% FA and was operated at an overall organic content. c) Analysis of basic negative ion employing a C18 column with methanol and water as mobile phase that contained 6.5 mM Ammonium Bicarbonate at pH 8. d) negative ionization following elution from a HILIC column (Waters UPLC BEH Amide 2.1x150 mm, 1.7 µm) using a gradient consisting of water and acetonitrile with 10mM Ammonium Formate, pH 10.8. The MS analysis covered approximately 70-1000 m/z.

Metabolic compounds were identified by comparison to the Metabolon libraries of purified standards and recurrent unknown metabolites (Evans et al., 2009). The identification was based on retention index within a narrow RI window of the proposed identification, accurate mass match to the library +/- 10 ppm, and the MS/MS forward and reverse scores. The use of these three criteria distinguishes and differentiates biomolecules. Metabolon carries more than 3,300 commercially available purified standard compounds for determination of analytical characteristics. In addition, for structurally unnamed molecules, respective mass spectral entries have been added to the libraries for future identification.

Data from cell pellets and culture supernatants were analyzed separately. Raw intensity values were re-scaled for each identified metabolite by dividing them by the median intensity across samples. Missing values for a given metabolite and sample were imputed by assigning the minimum value for the metabolite across samples. The scaled and imputed data were Log_10_ transformed for subsequent analyses. Principal component analysis (PCA) was used to analyze the similarity of metabolic profiles between samples. For supernatant samples, secreted metabolites were identified by comparing the scaled and imputed intensities to the respective metabolites in media controls. A 1.5-fold increase in scaled intensities over media was used to define metabolites secreted. A similar 1.5-fold increase between an individual strain and the remaining 2 strains, or between strain consortia and the corresponding individual strains, was used to define uniquely secreted metabolites.

**REFERENCES**

Efsa Panel on Additives and Products or Substances Used in Animal Feed (Feedap), G.R., Gabriele Aquilina, Giovanna Azimonti, Vasileios Bampidis, Maria De Lourdes Bastos, Georges Bories, Andrew Chesson, Pier Sandro Cocconcelli, Gerhard Flachowsky, Jürgen Gropp, Boris Kolar, Maryline Kouba, Marta López‐Alonso, Secundino López Puente, Alberto Mantovani, Baltasar Mayo, Fernando Ramos, Maria Saarela, Roberto Edoardo Villa, Robert John Wallace, Pieter Wester, Boet Glandorf, Lieve Herman, Sirpa Kärenlampi, Jaime Aguilera, Montserrat Anguita, Rosella Brozzi, Jaume Galobart (2018). "Guidance on the characterisation of microorganisms used as feed additives or as production organisms". EFSA (European Food Safety Authority)).

Evans, A.M., Dehaven, C.D., Barrett, T., Mitchell, M., and Milgram, E. (2009). Integrated, Nontargeted Ultrahigh Performance Liquid Chromatography/Electrospray Ionization Tandem Mass Spectrometry Platform for the Identification and Relative Quantification of the Small-Molecule Complement of Biological Systems. *Analytical Chemistry* 81**,** 6656-6667.

Latorre, J.D., Hernandez-Velasco, X., Wolfenden, R.E., Vicente, J.L., Wolfenden, A.D., Menconi, A., Bielke, L.R., Hargis, B.M., and Tellez, G. (2016). Evaluation and Selection of *Bacillus* Species Based on Enzyme Production, Antimicrobial Activity, and Biofilm Synthesis as Direct-Fed Microbial Candidates for Poultry. *Front Vet Sci* 3**,** 95.

V.Mccleary, B. (1978). A simple assay procedure for β-d-mannanase. *Carbohydrate Research* 67 213-221.
